# Transoral endoscopic thyroidectomy vestibular approach as a novel technique for pediatric populations: Results from a single surgeon

**DOI:** 10.3389/fendo.2023.1177633

**Published:** 2023-06-02

**Authors:** Duy Quoc Ngo, Duong The Le, Quy Xuan Ngo, Quang Van Le

**Affiliations:** ^1^ Department of Head and Neck Surgery, Vietnam National Cancer Hospital, Hanoi, Vietnam; ^2^ Department of Oncology, Hanoi Medical University, Hanoi, Vietnam

**Keywords:** transoral thyroidectomy, TOETVA, transoral approach, thyroid surgery in children, pediatric population

## Abstract

**Background:**

The transoral endoscopic thyroidectomy vestibular approach (TOETVA) is increasingly being adopted worldwide because of its many advantages. However, there are few reports on the effectiveness and safety of TOETVA in children. In this study, we report the results of the application of TOETVA on 27 pediatric patients in Vietnam. To the best of our knowledge, this is also the largest sample size of the TOETVA technique performed by a single surgeon on pediatric patients worldwide. Patients and methods: From June 2020 to February 2022, we performed TOETVA on 27 pediatric patients (≤ 18 years old). The outcomes of the procedure were retrospectively reviewed.

**Results:**

Our study was conducted on 27 pediatric patients, of whom 24 were female (88.9%). The mean age was 16.3 ± 2 (range 10-18). Fifteen patients had benign thyroid nodules with a mean nodule size of 31.6 ± 7.1 (range 20-50mm), and 12 patients had papillary thyroid carcinoma with a mean nodule size of 10.2 ± 5.6 (range 4-19mm). All 27 patients underwent successful TOETVA without any conversion to open surgery. The 15 patients with benign thyroid nodules had lobectomies with a mean operative time of 83.3 ± 10.5 (range 60-105 minutes). Among the 12 patients diagnosed with thyroid cancer, ten had a lobectomy, isthmusectomy, and central neck dissection, with a mean operative time of 89.8 ± 5.7 (range 80-100 minutes). The other two underwent total thyroidectomy with central lymph node dissection with a mean operative time of 132.5 minutes. The mean hospital stay was 4.7 ± 0.9 (range 3-7 days). No patient had permanent complications, such as hypocalcemia, recurrent laryngeal nerve injury, or mental nerve injury. The rates of temporary recurrent laryngeal nerve injury and mental nerve injury were 3.7% and 11.1% respectively.

**Conclusions:**

TOETVA may be a feasible and safe surgical method for children with thyroid disease. However, we recommend that only high-volume thyroid surgeons with experience in TOETVA should perform TOETVA on the pediatric population.

## 1 Introduction

Thyroid diseases in children, including hypothyroidism, hyperthyroidism, benign thyroid nodules, and thyroid gland cancer, are uncommon (1). Thyroid cancer, mainly well-differentiated thyroid carcinoma, accounts for 1.5% of all cancers in this age group and is increasing in incidence (2, 3). As the prognosis for well-differentiated thyroid carcinoma in the pediatric population is excellent, it is essential to consider quality of life in this group (4). In the surgical treatment of thyroid disorders in children, open thyroidectomy is still the primary method, but this method leaves a scar on the front neck area. Some studies have shown that neck scars after open thyroidectomy may affect patient confidence and reduce their quality of life, especially in the pediatric population (5, 6). Many new approaches are being used that limit scarring in the anterior neck area, such as minimally invasive video-assisted thyroidectomy (MIVAT) and the transaxillary, bilateral axillobreast, and retroauricular methods. Each method has advantages and disadvantages, and studies on using these methods with pediatric patients are limited.

The transoral endoscopic thyroidectomy vestibular approach (TOETVA) is a new surgical method with many advantages, such as a short approach, the ability to access both thyroid lobes, the ability to remove central neck lymph nodes, and, in particular, the absence of scars on the skin, giving especially aesthetic results (7–9). Therefore, TOETVA is increasingly being used worldwide. However, there have been few reports on the effectiveness and safety of TOETVA in pediatric patients. Only three studies have been published worldwide, reporting on 53 pediatric patients who underwent TOETVA (10–12). In Vietnam, TOETVA was first implemented in 2018 and is now a routine technique in our hospital (13). In this study, we report the results of the TOETVA method on 27 pediatric patients. To the best of our knowledge, this is the largest sample size for the TOETVA technique performed by a single surgeon on pediatric patients worldwide to date.

## 2 Patient and methods

Assessed according to American Thyroid Association (ATA) guidelines, patients ≤ 18 years were included in this study (2). Between June 2020 and February 2022, 27 patients were admitted to the Department of Head and Neck Surgery and selected for a transoral approach. These patients had a preoperative assessment including thyroid hormonal results, neck ultrasound examination, and fine needle aspiration (using the Bethesda System for Reporting Thyroid Cytopathology - TBSRTC) (14). The open surgery and transoral endoscopic approaches were carefully explained to patients and their parents. Written informed consent and patient parental informed consent were obtained for all participants. The study was approved by the ethical research committee at our hospital in 2020.

Inclusion criteria for TOETVA techniques were: (1) thyroid benign nodule ≤ 6 cm in size; (2) suspicious tumor or well-differentiated thyroid cancer at cT1N0M0 stage; (3) size of one thyroid lobe ≤ 10 cm estimated by ultrasound; (4) patient ≤18 years old who had a desire to avoid a cervical scar (9).

Contraindications for TOETVA included: (1) radiation of the head and neck prior to surgery; (2) previous surgery in the neck or submental area; (3) inability to perform surgery; (4) oral cavity infection; (5) uncontrolled hyperthyroidism; (6) patients under 10 years old or weighing ≤ 30 kg (9).

### 2.1 Surgical technique

**Figure 1:**
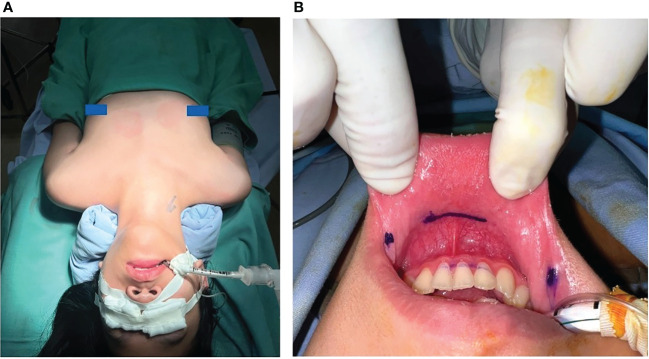
**(A)** Patient was placed in the supine position with the neck extended; **(B)** Three incisions were made in the oral vestibule.

**Figure 2:**
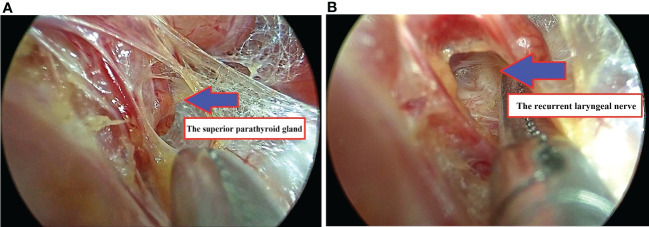
**(A)** The upper parathyroid gland was identified; **(B)** The right recurrent laryngeal nerve was exposed.

In brief, the patients were placed in the supine position with the neck extended (**Figure 1**). After intubation, we injected about 10 mL of dilute epinephrine-saline solution (1:200 000) into the lower lip to the tip of the chin. Then, three incisions were made in the oral vestibule to insert the endoscopic 5-10 mm trocars (**Figure 1**). In order to insert a 30-degree endoscope, a 5 mm trocar is recommended for the central trocar, especially for pediatric patients. A laparoscopic cautery hook and ultrasonic scalpel expanded the working space further caudally to reach the sternal notch inferiorly and the sternocleidomastoid laterally. The strap muscles were divided in the midline to expose the thyroid gland. Then, the right ipsilateral strap muscle was laterally retracted by a transcutaneous 3/0 silk suture to enhance the working space. The pyramidal lobe was dissected and separated from the trachea. The isthmus was then divided, and the superior thyroid vessels were dissected and divided using an energy device. The upper parathyroid gland was then identified, followed by the visual identification of the recurrent laryngeal nerve (RLN) (**Figure 2**). The thyroid lobe was dissected from the trachea and the RLN while preserving the lower parathyroid. Central neck dissection was performed for cancer patients while preserving the lower parathyroid gland, and the lymph-adipose tissue was retrieved en bloc. The specimen was put into an endocatch bag and removed *via* the central incision. The strap muscles were closed with a 3-0 absorbable suture. The trocars were removed, and the incisions were closed in two layers with absorbable interrupted sutures.

## 3 Results

Our study was conducted on 27 pediatric patients, of whom 24 were female (88.9%). The mean age was 16.3 ± 2 (range 10-18). None of the patients had a family history of thyroid cancer.Fifteen patients had benign thyroid nodules with a mean nodule size of 31.6 ± 7.1 (range 20-50mm), and 12 patients had papillary thyroid carcinoma with a mean nodule size of 10.2 ± 5.6 (range 4 to 19mm) (**Table 1**).

**Table 1:** Preoperative data summary of the study’s cohort.

Parameter	n (%)
Age (mean ± SD), years	16.3 ± 2 (ranged 10 – 18)
Sex	
Female	24 (88.9%)
Male	3 (11.1%)
Location	
Right lobe	16 (59.3%)
Left lobe	11 (40.7%)
Nodule size (mean ± SD), mm	
Benign group	31.6 ± 7.1 (ranged 20-50)
Thyroid group	10.2 ± 5.6 (ranged 4-19)
Fine Needle Aspiration	
Bethesda II	15 (55.6%)
Bethesda III	1 (3.7%)
Bethesda IV	0
Bethesda V	6 (22.2%)
Bethesda VI	5 (18.5%)
Pathological results	
Benign	15 (55.6%)
Papillary thyroid carcinoma	12 (44.4%)
Types of thyroid surgery	
Lobectomy	15 (55.6%)
Lobectomy, isthmusectomy, and central neck dissection	10 (37.0%)
Total thyroidectomy and central neck dissection	2 (7.4%)
Characteristics of cancer patients	
pT1a stage	6 (50%)
pT1b stage	6 (50%)
pN0 stage	5 (41.7%)
pN1a stage	7 (58.3%)
The number of harvested lymph nodes (mean ± SD)	4.2 ± 1.9 (ranged 2-9)

All 27 patients underwent successful TOETVA surgery by a single surgeon at our hospital without any conversion to open surgery. The 15 patients with benign thyroid nodules had lobectomies with a mean operative time of 83.3 ± 10.5 (range 60-105 minutes). Of the 12 patients diagnosed with thyroid cancer, ten had a lobectomy, isthmusectomy, and central neck dissection with a mean operative time of 89.8 ± 5.7 (range 80-100 minutes). The other two had total thyroidectomy with central lymph node dissection with a mean operative time of 132.5 minutes. No patients required a postoperative drain. The mean hospital stay was 4.7 ± 0.9 (range 3-7 days). Most patients could eat porridge or milk 6-8 hours after surgery and resume normal activities from the first postoperative day.

**Figure 3:**
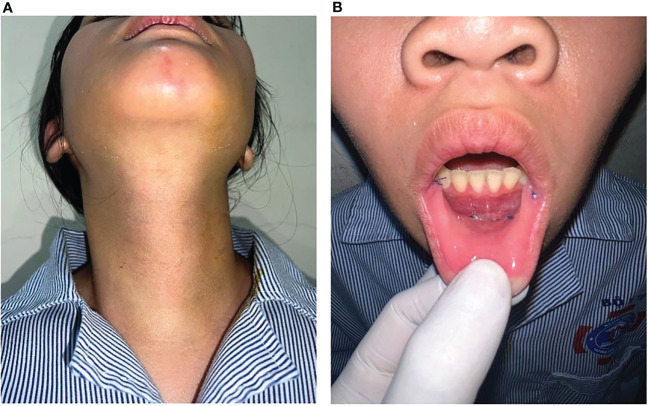
**(A)** The picture of anterior neck three days after surgery; **(B)** The patient’s vestibular area three days after surgery.

Postoperative follow-up and evaluation after one year showed no permanent complications, including hypocalcemia, RLN injury, or mental nerve injury. Temporary recurrent laryngeal nerve injury occurred in 1/27 (3.7%) patients, with hoarseness starting the second day after surgery and full recovery after three months. No temporary hypocalcemia was recorded after total thyroidectomy. After surgery, three patients developed numbness in the chin and lower lip but fully recovered after one month (**Table 2**). No patients had bleeding, infection, or fluid accumulation after surgery (**Figure 3**).

**Table 2:** Detailed summary of patients with complications in the cohort.

Parameter	Results
Operative time (mean ± SD), mins	
Lobectomy	83.3 ± 10.5 (ranged 60-105)
Lobectomy, isthmusectomy, and central neck dissection	89.8 ± 5.7 (ranged 80-100)
Total thyroidectomy and central neck dissection (2 patients)	132.5 (120,145)
Complications (n, %)	
Postoperative bleeding	0
Conversion to open surgery	0
Temporary recurrent laryngeal nerve injury	1 (3.7%)
Permanent recurrent laryngeal nerve injury	0
Temporary hypocalcemia	0
Permanent hypocalcemia	0
Temporary mental nerve injury	3 (11.1%)
Permanent mental nerve injury	0
The hospital stay (mean ± SD), days	4.7 ± 0.9 (ranged 3-7)

In the 12 cancer patients, postoperative pathological results showed papillary thyroid carcinoma with tumor sizes of less than 2cm; 50% were T1a and 50% T1b. Of these, ten patients (83.3%) were indicated for thyroid lobectomy, isthmusectomy, and central neck dissection and two patients for total thyroidectomy with bilateral central neck dissection. 7/12 (58.3%) patients had central lymph node metastases; the mean number of lymph nodes removed was 4.2 ± 1.9 (range 2-9 lymph nodes).

## 4 Discussion

Thyroid cancer in children (patients ≤ 18 years old) mainly takes the form of well-differentiated thyroid carcinoma (2). This is a rare disease that accounts for 1.5% of all childhood cancer cases, with an annual incidence rate of 4.8-5.9 cases per million children, although the rate is increasing (2, 3). Well-differentiated thyroid cancer in children has a good prognosis. A study by Hogan et al. (2009) on 1,753 pediatric patients with well-differentiated thyroid cancer found 5-year, 10-year, and 30-year survival rates of 98%, 97%, and 92%, respectively (15). Another study, of 227 children with well-differentiated thyroid cancer, reported 10-year, 20-year, and 40-year survival rates of 99.3%, 99.3%, and 96.5%, respectively (16). Thus, in addition to curing the disease, choosing a treatment method that helps improve children’s long-term quality of life is essential (4).

In surgery for pediatric thyroid diseases, open thyroidectomy is still the primary method, but it leaves a scar on the front neck area that can affect children, impacting quality of life and increasing the risk of depression (5, 17–19). Children are vulnerable to emotional distress when there is a visible scar on the front neck area, particularly with changes in psychosocial development during adolescence. In addition, the risk of hypertrophic neck scars in children is higher than in adults (20).

TOETVA is increasingly being applied worldwide because of the advantages it offers over other approaches. However, there have been very few reports on the use of this method in pediatric patients. In 2021, a study from six large thyroid centers with extensive experience with TOETVA in Brazil, Israel, Italy, Korea, Thailand, and the United States recorded 48 pediatric patients who had been subject to the procedure (10). The initial results showed TOETVA to be a feasible and safe surgical method for children with thyroid diseases. This study is the largest report on the TOETVA method in pediatric patients worldwide to date.

Our study involved 27 pediatric patients with a mean age of 16.2 ± 2 (range 10-18). Most of the patients were females (88.9%). The age range in our study is similar to that of other studies in pediatric patients, such as Lee’s study (2021) with a mean age of 16.9 ± 2.3 (range 8-19 years old) (21) and Oded Cohen’s study with a mean of 16 years old (range 10-17 years old) (10). Of the 27 patients in our study, 15 had benign thyroid nodules in a thyroid lobe (55.6%), and 12 were diagnosed with papillary thyroid carcinoma (44.4%). Of the 12 patients with cancer, two underwent total thyroidectomy with central neck dissection due to contralateral nodal involvement, and the other ten underwent thyroid lobectomy with central neck dissection as they had tumors <1 cm, cN0M0 stage, and no contralateral thyroid lobe involvement. All the cancer patients underwent prophylactic central neck dissection because of the high rate of central lymph node metastasis in the N0 group; this improves disease-free survival in pediatric patients (22, 23). In the 12 patients with thyroid cancer, the mean number of central neck lymph nodes removed was 4.2 ± 1.9 (range 2-9); 7 patients had pN1a lymph node metastasis after surgery (58.3%). With the transoral approach, surgeons can efficiently perform central neck lymph node dissection, which is one of the main advantages of this approach compared to other approaches (24). The ATA recommends total thyroidectomy for pediatric patients with thyroid cancer because the rate of bilateral tumors is up to 30%, and the frequency of multifocal disease is high (2, 25). However, after surgery, patients must have supplementary thyroid hormones for the rest of their life. Total thyroidectomy also increases the risk of complications, such as temporary or permanent hypoparathyroidism and RLN injury, which can affect the quality of life of pediatric patients. Therefore, some authors support thyroid lobectomy and isthmusectomy in cases of low-risk thyroid cancer, such as a cT1N0M0 stage, and no history of previous neck irradiation (16, 26–28).

In our study, the mean size of the benign thyroid nodules was 31.6 ± 7.1 (range 20-50mm), while the average size of cancerous nodules was 10.2 ± 5.6 (range 4-19mm). These results are similar to a study by Oded Cohen, which reported a mean nodule size of 3.2 ± 1.5 cm for 48 cases of TOETVA in children, with a mean malignant nodule size of 1.4 ± 0.4 cm (10). Nodule size is an essential consideration in pediatric patients, as their anatomy is generally smaller than adults, especially in children under 15 years old. For adults, TOETVA is indicated for benign nodules ≤ 6 cm and malignant nodules ≤ 2 cm (13). However, for pediatric patients, especially those under 15 years old, selecting patients with smaller nodules for TOETVA is recommended to ensure the safety of the surgery. In our study, the largest nodule size was 5 cm, but the patient was 17 years old with an anatomy similar to an adult, and we had experience performing TOETVA on nearly 500 patients before this surgery, making the procedure safe with no complications.

All the patients were successfully operated on using the TOETVA technique without any conversion to open surgery. The surgical time in our study for patients with lobectomy, lobectomy with central neck node dissection, and total thyroidectomy with central neck node dissection were 83.3 ± 10.5 (range 60-105 minutes), 89.8 ± 5.7 (range 80-100 minutes), and 132.5 (range 120-145 minutes), respectively. Our surgical time was shorter than in Oded Cohen’s study, with an average operative time of 100 minutes and 200 minutes for the lobectomy and total thyroidectomy groups, respectively (10). This difference may be due to our gaining extensive experience with TOETVA on adult patients before applying it to pediatric patients, resulting in shorter surgical times. In addition to endoscopic surgery, many authors worldwide have also used the Da Vinci robotic system in thyroid surgery in pediatric populations. However, robotic surgery often takes considerably longer, as can be seen in Lee’s study (robotic axillary surgery) with a mean surgical time of 171.2 ± 101.7 minutes, and Li’s 2022 study (bilateral axillobreast robotic surgery for total thyroidectomy) with an average surgical time of 217.5 minutes (29). Robotic surgery usually takes longer as the surgeon needs time to perform the docking process of the robotic arms.

Thyroidectomy can lead to dangerous complications, especially in children. A study conducted on 464 children who underwent open thyroidectomy showed that the most common complications were temporary hypoparathyroidism (37%) and temporary RLN injury (2.37%) (30). Permanent hypoparathyroidism or RLN injury rates were very low (less than 1%). With TOETVA, Cohen reported temporary hypoparathyroidism and RLN injury rates in 48 children of 33% and 1.6%, respectively (10). No patients experienced permanent RLN injury or hypoparathyroidism. Our study reported temporary hoarseness in 1 out of 27 patients (3.7%), who recovered after 3 months, with no permanent hoarseness observed. The temporary hoarseness occurred in a patient with papillary thyroid carcinoma in the posterior location on the right lobe; the RLN may have been temporarily damaged during dissection. After performing TOETVA on approximately 500 patients, we have found that tumors in the posterior aspect of the thyroid gland can cause more difficulty in exposing and preserving the nerve. Therefore, surgeons should be aware of the tumor location on ultrasound and choose appropriate patients for TOETVA, not only in children but also in other age groups. Nerve integrity monitors (NIM) can be used to identify and preserve the RLN during surgery to reduce the rate of hoarseness, especially in children. However, our study did not use this technique because it is not widely available in Vietnam. When performing the transoral approach, the RLN is followed from top to bottom and not the reverse; there is concern about the increased risk of RLN injury during TOETVA compared to open thyroidectomy since it can present several branches. During TOETVA, the RLN is usually identified at its insertion site and released first after the division of Berry’s ligament. This approach can minimize the risk of RLN injury.

We have extensive experience in thyroid surgery (2,000-3,000 open surgeries and 300-400 TOETVA surgeries per year), making nerve identification and preservation relatively straightforward. Only two patients in our study underwent total thyroidectomy, and no patients experienced temporary or permanent hypoparathyroidism. However, Cohen and colleagues reported a temporary hypoparathyroidism rate of up to 33% (10). This difference may be due to the small number of patients who underwent total thyroidectomy in our study compared to Cohen’s study. However, neither study had patients with permanent hypoparathyroidism or RLN injury.

Specific complications related to the transoral approach include injury to the mental nerves, infection, and skin flap perforation. In our study, 3/27 (11.1%) patients experienced temporary numbness in the chin and lower lip area after surgery but recovered within one month; no patient had an infection. In Oded Cohen’s study, the temporary mental nerve injury rate was 4.2%, with no patients experiencing permanent mental nerve injury or infection (10). Compared to other groups, the incidence of complications in pediatric patients is no different. According to a pooled analysis of 1,887 patients, the temporary and permanent rates of hypocalcemia are 0.94-22.2% and 1.33-2.22%, respectively; the temporary and permanent rates of hoarseness are 1.9-8.8% and 0.59-1.42%, respectively. The rate of mental nerve injury is 5.8%, and the infection rate is 0.64% (12/1887) (31). Our study did not report any unusual complications, such as skin perforation, burn, trauma, or bruising related to TOETVA. Therefore, TOETVA in pediatric patients may be a safe and effective technique. Of particular note, all the patients in our study were delighted with the cosmetic results after surgery; this outcome is an important advantage of the oral approach compared to other approaches, especially in pediatric patients.

Endoscopic thyroidectomy in children presents more challenges than in other age groups due to the smaller anatomical structures. Therefore, based on experience with TOETVA on 500 adult cases and 27 pediatric cases, we recommend selecting pediatric patients for TOETVA who are ≥10 years old and weigh ≥30 kg to ensure minimal size difference in anatomical structures. In cases where the patient is <10 years old or weighs <30 kg, implementing TOETVA may be more difficult given the narrow surgical fields, which can increase the risk of complications. However, specialized equipment, such as smaller trocars and endoscopes, may allow the use of this technique in younger or smaller patients.

Our study has limitations, such as a small sample size, retrospective design, and short follow-up time, particularly for pediatric patients with thyroid cancer. In the future, we plan to continue expanding our patient cohort and follow-up period to evaluate fully the role of TOETVA in treating pediatric thyroid diseases.

## 5 Conclusion

TOETVA may be a feasible and safe surgical method for children with thyroid diseases. However, we highly recommend that only high-volume thyroid surgeons with experience in TOETVA should perform TOETVA on the pediatric population.

## Data availability statement

The original contributions presented in the study are included in the article/supplementary material. Further inquiries can be directed to the corresponding author.

## Ethics statement

The studies involving human participants were reviewed and approved by Vietnam National Cancer Hospital. Written informed consent to participate in this study was provided by the participants’ legal guardian/next of kin. Written informed consent was obtained from the individual(s), and minor(s)’ legal guardian/next of kin, for the publication of any potentially identifiable images or data included in this article.

## Author contributions

DN: Study design, performance of the study, data collection, statistical analysis, interpretation, and manuscript writing. DN, QN, DL: Data analysis and interpretation. DL: Performance of the study and data collection. DN, DL and QL: Performance of the study. All authors participated in the critical review and approval of the final manuscript. All authors contributed to the article and approved the submitted version.
